# Comparative evaluation of generative AI models for chest radiograph report generation in the emergency department

**DOI:** 10.1007/s00330-026-12648-8

**Published:** 2026-06-10

**Authors:** Woo Hyeon Lim, Ji Young Lee, Jong Hyuk Lee, Saehoon Kim, Hyungjin Kim

**Affiliations:** 1 Department of Radiology, Seoul National University Hospital, Jongno-gu, Korea; 2 Department of Radiology, Seoul National University College of Medicine, Jongno-gu, Korea; 3 Soombit.ai, Bundang-gu, Korea

**Keywords:** Diagnostic imaging, Generative artificial intelligence, Natural language processing, Radiography (Thoracic), Radiologists

## Abstract

**Objectives:**

To benchmark medical image-specific vision–language models (VLMs) against real-world radiologist-written reports, focusing on diagnostic quality, clinical acceptability, hallucinations, and language clarity.

**Materials and methods:**

This retrospective study included adult patients who presented to the emergency department of a tertiary center between January 2022 and April 2025 and underwent same-day chest radiograph (CXR) and CT for febrile or respiratory symptoms. Reports from five VLMs (AIRead, Lingshu, MAIRA-2, MedGemma, and MedVersa) and radiologist-written reports were randomly presented and blindly evaluated by three thoracic radiologists using four criteria: RADPEER, clinical acceptability, hallucination, and language clarity. Comparative performance was assessed using generalized linear mixed models, with radiologist-written reports treated as the reference. Finding-level analyses were also performed with CT as a reference.

**Results:**

A total of 478 patients (median age, 67 years [interquartile range, 50–78]; 282 males [59.0%]) were included. AIRead demonstrated the lowest RADPEER 3b rate (5.3% [76/1434] vs radiologists 13.9% [200/1434]; *p* < 0.001) and the highest clinical acceptability (84.5% [1212/1434] vs radiologists 74.3% [1065/1434]; *p* < 0.001), with hallucination rates comparable to radiologists (0.3% [4/1425]) vs 0.1% [1/1425]; *p* = 0.21). Other VLMs showed higher disagreement (16.8–43.0%; *p* < 0.05), lower acceptability (41.1–71.4%; *p* < 0.05), and more frequent hallucinations (5.4–17.4%; *p* < 0.05). Language clarity was higher for several VLMs (AIRead, Lingshu, and MedVersa) than for radiologist-written reports (82.9–88.4% [1189–1268/1434] vs 78.1% [1120/1434]; *p* < 0.05). Finding-level analyses showed substantial variability in sensitivity across VLMs for common thoracic findings.

**Conclusion:**

Medical VLMs for CXR report generation exhibited variable performance in report quality and diagnostic measures.

**Key Points:**

***Question*** *How do medical VLMs perform compared with radiologist-written reports regarding diagnostic quality, clinical acceptability, hallucinations, and language clarity for CXRs*?

***Findings*** *One non-open-source VLM achieved the lowest RADPEER 3b rate and highest acceptability with radiologist-level hallucinations, whereas other models showed inferior performance*.

***Clinical relevance*** *VLMs may support automated preliminary CXR reporting and serve as adjunct tools to enhance workflow efficiency and consistency. Although performance varied widely, carefully developed models approached radiologist-level quality, supporting continued refinement and targeted clinical integration*.

**Graphical Abstract:**

**Figure:**
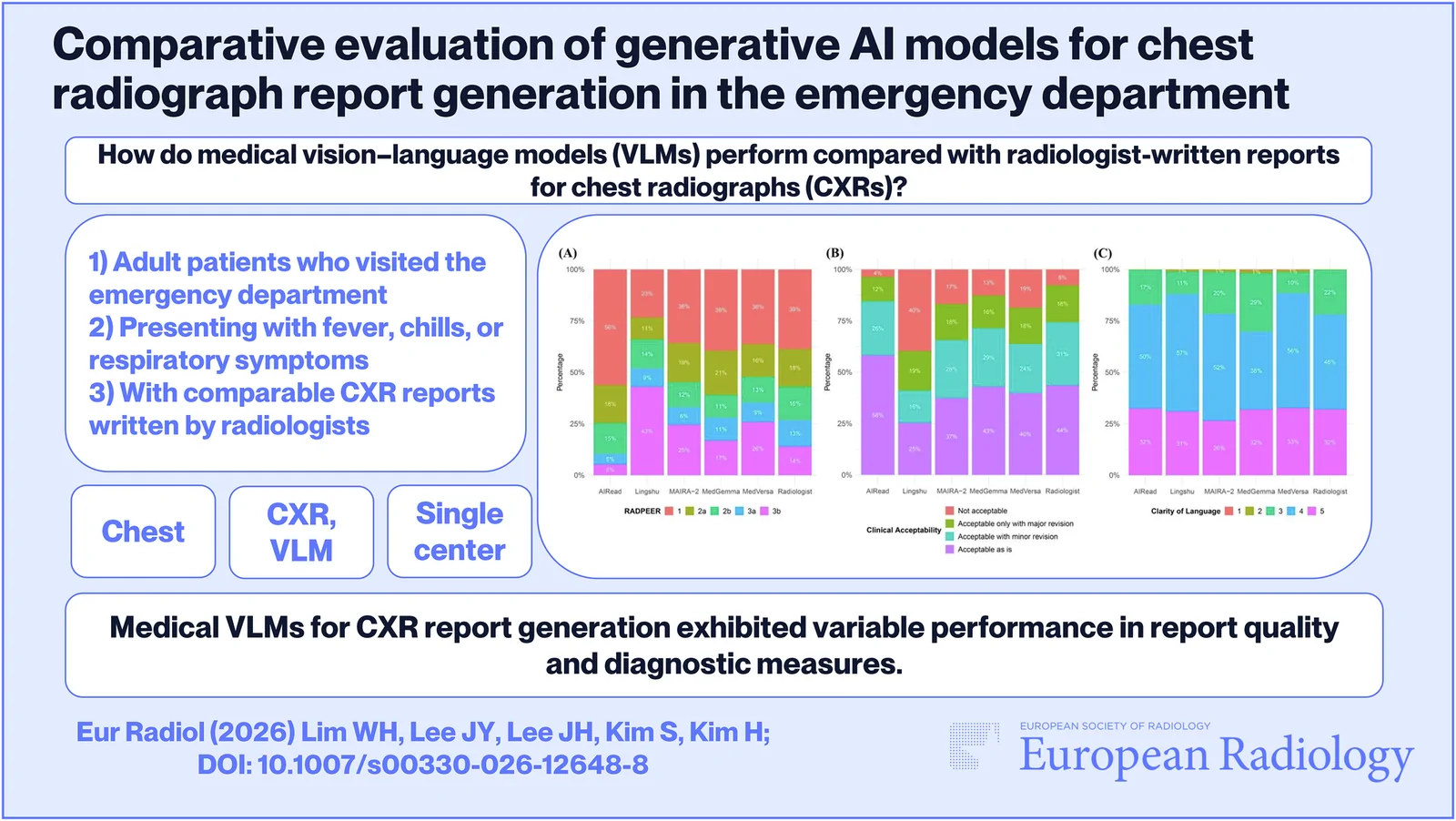

## Introduction

Over the past decade, artificial intelligence (AI)-based approaches, including deep learning, have shown promise in detecting and localizing abnormalities in chest radiographs (CXRs) [1]. More recently, the emergence of vision–language models (VLMs), which jointly process images and text, has brought attention to radiologic report generation [1]. This is particularly relevant considering the global shortage of radiologists, which is increasingly strained by the growing demand and clinical importance of imaging studies [2–4]. VLM-based report generation has therefore been proposed as a potential solution for producing timely and clinically useful reports in settings with limited radiologist availability [1].

Previous studies have explored the performance of AI in CXR report generation, most often evaluating outputs with semantic similarity measures or automated metrics based on label extraction [5–8]. However, assessing the clinical utility and quality of AI-generated reports requires a multifaceted approach to accurately determine the readiness of VLMs for clinical translation [9, 10]. For instance, one prior study employed multidimensional assessments including clinical acceptability, expert agreement, and overall report quality [10]. Key dimensions of VLM-generated report evaluation should encompass diagnostic performance (report-level and finding-level), clinical acceptability (i.e., whether generated reports are suitable for clinical use with only minor revisions), absence of hallucination, and linguistic clarity [10, 11].

Medical image-specific VLMs can internalize radiology priors beyond what generalist models acquire from natural images and captions [10–12]. Although medical VLMs have demonstrated promising performance in isolation [13–16], the literature currently lacks a systematic, standardized comparison across multiple medical VLMs for the specific task of CXR report generation. It is essential to compare model-generated reports under a unified framework and identical experimental conditions.

In this retrospective study, we address this gap by conducting a systematic head-to-head benchmarking of medical image-specific VLMs for CXR report generation. We performed a multidimensional comparison of five recently released models against real-world radiologist-written reference reports, using CXRs obtained from patients presenting to the emergency department.

## Materials and methods

This retrospective study was approved by the Institutional Review Board of Seoul National University Hospital (No. H-2505-199-1646), with a waiver of informed consent.

### Patients

Adult patients presenting to the emergency department of a tertiary referral center between January 2022 and April 2025 were retrospectively identified. Eligible patients were those who underwent initial frontal CXR (anteroposterior or posteroanterior view) and chest CT on the same day, with CT serving as the reference standard for finding-level assessment. Among these, patients presenting with fever and/or chills or respiratory symptoms—including dyspnea, cough with or without sputum, blood-tinged sputum or hemoptysis, and chest discomfort or pain—were included. The following patients were excluded: (1) those whose CXR reports contained comparison statements, (2) those whose reports were not available, and (3) those whose reports relied heavily on clinical information rather than imaging findings.

### Data collection

From the electronic medical records, we collected patient demographics, radiologist-written CXR reports, the interval between CXR and CT scan, chief complaints, and comorbidities. Digital Imaging and Communications in Medicine files of CXRs were exported from the Picture Archiving and Communication System. CXRs between January 2022 and February 2024 were primarily interpreted by radiology residents, whereas those between March 2024 and April 2025 were interpreted by board-certified radiologists. The latter period coincided with a nationwide strike by medical students, residents, and fellows, which shifted the emergency department workload exclusively to board-certified radiologists [17, 18].

### CXR report generation

Five medical image-specific VLMs were employed: AIRead (Soombit.ai), Lingshu (Alibaba), MAIRA-2 (Microsoft Research), MedGemma (Google DeepMind), and MedVersa (Harvard University) [10, 13–16]. Among these, the latter four models are open-source. For each model, CXR reports were generated from the corresponding Digital Imaging and Communications in Medicine files, without access to clinical information or comparison images. Model inference was performed on a single NVIDIA H100 graphics processing unit running CUDA 12.4, without fine-tuning or transfer learning. Details of each model, including availability and the prompts used, are provided in Supplementary Text 1.

### Evaluation of VLM-generated CXR reports

VLM-generated CXR reports were evaluated using two approaches: (1) a multireader study assessing the diagnostic quality of free-text reports based on four criteria (RADPEER score, clinical acceptability, presence of hallucinations, and language clarity) [10, 19], and (2) finding-level diagnostic sensitivity and specificity.

#### Report-level diagnostic quality

The RADPEER scoring system was originally developed by the American College of Radiology as a peer review tool for quality assurance [19]. It uses a three-point scale: a score of 1 indicates complete agreement with the original interpretation, while scores of 2 (understandable miss) and 3 (unacceptable miss) indicate increasing levels of disagreement. Subcategories “a” and “b” further classify findings as clinically insignificant or clinically significant, respectively. The study outcomes were defined as the proportions of reports with a score of 3b (unacceptable, clinically significant miss) and with scores of 2b or 3b combined (any clinically significant miss [i.e., stricter criterion]).

Clinical acceptability was evaluated on a four-point scale that considered the presence of detection errors (e.g., false negatives, false positives, incorrect laterality) and the extent of editing required for clinical use, complementing the RADPEER system [11]. In this scale, a score of 1 denoted a report that was not acceptable because of detection errors for clinically referable abnormalities. A score of 2 denoted a report acceptable after major revision due to fewer critical detection errors or the need for extensive edits. A score of 3 denoted a report acceptable only with minor revision, being generally accurate but requiring limited edits. Finally, a score of 4 denoted a report acceptable as is, being accurate, clear, and clinically actionable without edits. The study outcome was the proportion of clinically acceptable reports, defined under two thresholds: (1) the standard criterion, encompassing reports acceptable as is or after minor revision, and (2) the stringent criterion, limited to reports acceptable as is.

Hallucination was defined as the inclusion of information that could not be derived from a single frontal CXR. Examples included references to a lateral view, patient history, comparison studies, or measurements (such as lesion size or distance) not supported by the study setting and models. Language clarity was evaluated as a measure of readability using a five-point scale (1, unacceptable; 2, poor; 3, moderate; 4, good; 5, excellent). The study outcome was the proportion of reports rated 4 or 5.

This evaluation framework was applied to reports generated by the five VLMs, as well as to radiologist-written reports. Three board-certified, fellowship-trained thoracic radiologists (J.H.L., 4 years of post-fellowship experience; W.H.L., 2 years; J.Y.L., 1 year) independently evaluated, for each case, all six reports (five VLM-generated and one radiologist-written) with access to the original CXR images, using a web-based assessment platform in a randomized, blinded manner. All assessments were performed in a single session without a washout period. The criteria are summarized in Supplementary Table 1.

#### Per-patient finding-level analyses

Finding-level sensitivity and specificity were measured for both VLM-generated and radiologist-written CXR reports, with same-day chest CT as the reference. CT scans were reviewed for the presence of predefined abnormalities: aortic aneurysm (≥ 50 mm for the ascending aorta, ≥ 40 mm for the descending aorta), cardiomegaly (CT cardiothoracic ratio > 0.5), cavity, emphysema, hilar mass, lung opacity (consolidation or ground-glass opacity), mediastinal mass, miliary nodules, non-calcified nodule (≥ 4 mm) or mass, pericardial effusion, pleural effusion, pneumomediastinum, pneumoperitoneum, pneumothorax, and reticular opacity [20–22]. Each CT was reviewed by one of three board-certified thoracic radiologists after completion of the reader study.

Then, a previously developed labeler was used to extract the presence or absence of individual findings from the CXR reports (Supplementary Text 2). Briefly, CheXGPT, a Bidirectional Encoder Representations from Transformers-based model trained on 3 million CXR reports, was used [23].

### Statistical analyses

The normality of continuous variables was assessed using the Shapiro–Wilk test. Continuous variables were presented as mean ± standard deviation or as median with interquartile range (IQR), depending on distribution.

The proportions of report quality measures were analyzed using generalized linear mixed-effects models with a logit link. Report source (VLMs or radiologists) was included as a fixed effect, using radiologists as the reference category, while reader and case were modeled as random intercepts. Cases in which original reports contained measurements were excluded from the hallucination analysis. Subgroup analyses were performed, stratified by sex (male vs female), age (≥ 65 vs < 65 years), and the expertise level of the interpreting radiologist (resident vs board-certified). For finding-level analyses, sensitivity and specificity with 95% confidence intervals were calculated for each report source and for each abnormality, using CT as the reference standard.

All statistical analyses were performed using R (version 4.1.2) by W.H.L., and a two-sided *p* < 0.05 was considered statistically significant.

## Results

### Patients

**Fig. 1:**
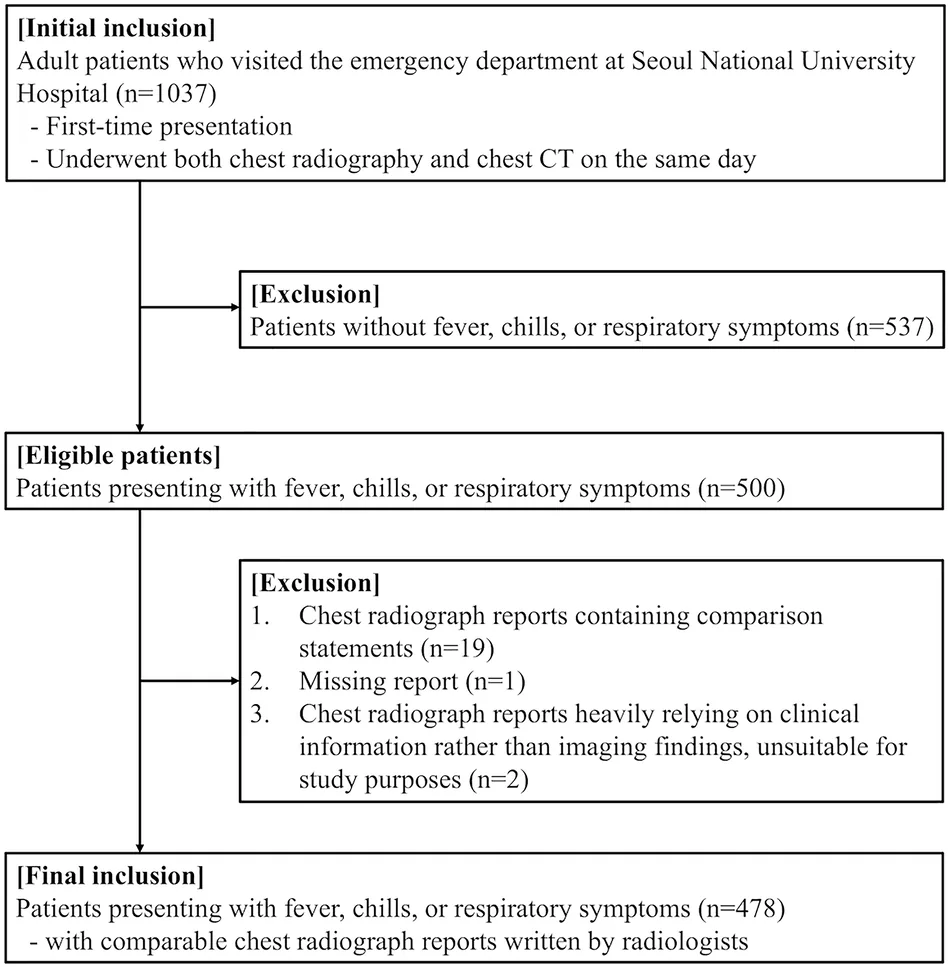
Flow diagram of patient inclusion and exclusion

Of the 1037 adult patients who presented to the emergency department and underwent initial CXR and CT, the following were excluded: (1) patients without fever, chills, or respiratory symptoms (*n* = 537), (2) patients whose CXR reports contained comparison statements (*n* = 19), (3) patients with missing reports (*n* = 1), and (4) patients whose reports relied heavily on clinical information rather than imaging findings (*n* = 2). A total of 478 patients (median age, 67 years [IQR, 50–78]; 282 male [59.0%]) were included in the final analyses (Fig. 1).

The most common chief complaint was dyspnea (47.7% [228/478]), followed by fever and/or chills (23.6% [113/478]) (Table 1). Malignancy was present in 5.4% (26/478) of patients (lung cancer, *n* = 9; other solid organ malignancy, *n* = 12; hematologic malignancy, *n* = 5), and diffuse parenchymal lung disease was identified in 3.1% (15/478; chronic obstructive pulmonary disease, *n* = 10; interstitial lung disease, *n* = 5) (Table 1). Among the included CXRs, 32.6% of patients (156/478) were interpreted as normal (i.e., no abnormal findings), whereas the remaining 67.4% (322/478) had at least one abnormality.

**Table 1:** Characteristics of study patients

Variable	No. of patients (*n* = 478)
Age, years*	67 (50–78)
Sex	
Men	282 (59.0)
Women	196 (41.0)
Chest CT-CXR interval, days*	0 (0–0)
Projection	
Posterior–anterior	237 (49.6)
Anterior–posterior	241 (50.4)
Chief complaint	
Fever and/or chill	113 (23.6)
Dyspnea	228 (47.7)
Cough and/or sputum	43 (9.0)
Blood-tinged sputum or hemoptysis	32 (6.7)
Chest discomfort or pain	62 (13.0)
Comorbidity	
Lung cancer	9 (1.9)
Other solid organ malignancy	12 (2.5)
Hematologic malignancy	5 (1.0)
Chronic obstructive pulmonary disease	10 (2.1)
Interstitial lung disease	5 (1.0)
Heart failure	12 (2.5)
Hypertension	156 (32.6)
Diabetes mellitus	94 (19.7)
Stroke	15 (3.1)
Coronary artery disease	17 (3.6)

Data are presented as the number of patients (percentage), unless otherwise specified

*CT* computed tomography, *CXR* chest radiograph

* Data are presented as medians (IQR)

### Report-level diagnostic quality

The median word count was 5 words (IQR, 4–7) for radiologists, 10 (IQR, 4–15) for AIRead, 32 (IQR, 27–38) for Lingshu, 19 (IQR, 14–28) for MAIRA-2, 275 (IQR, 242–321) for MedGemma, and 46 (IQR, 32–66) for MedVersa.

**Fig. 2:**
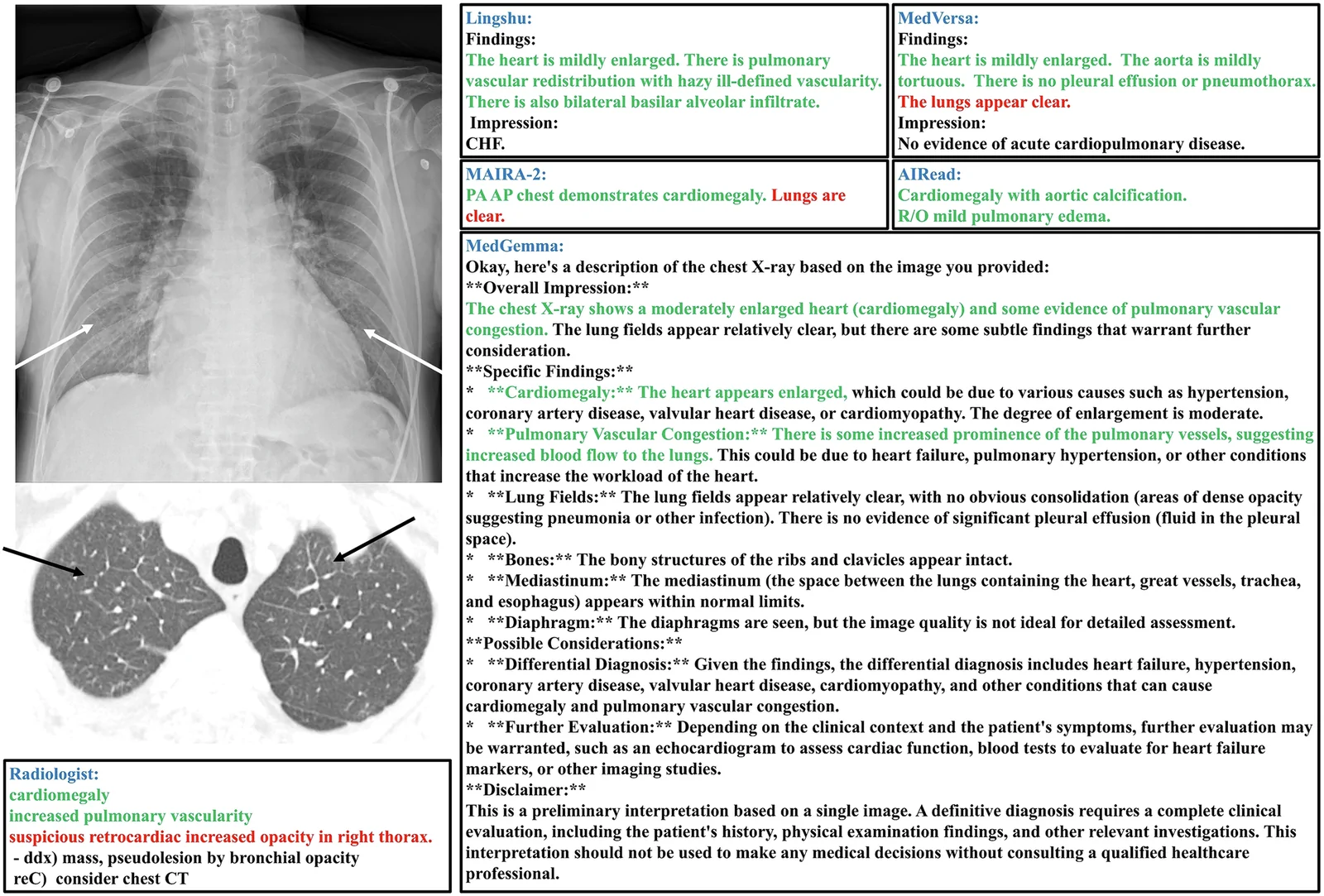
Representative CXRs and reports of true-positive cases. A 79-year-old male with dyspnea. Frontal CXR shows increased pulmonary vascularity with equivocal peribronchial opacities (white arrows); CT confirmed interlobular septal thickening (black arrows). Some report sources suggested pulmonary congestion/edema, whereas MAIRA-2 and MedVersa failed to report these findings. Green = true positive/true negative; red = false positive/false negative

**Fig. 3:**
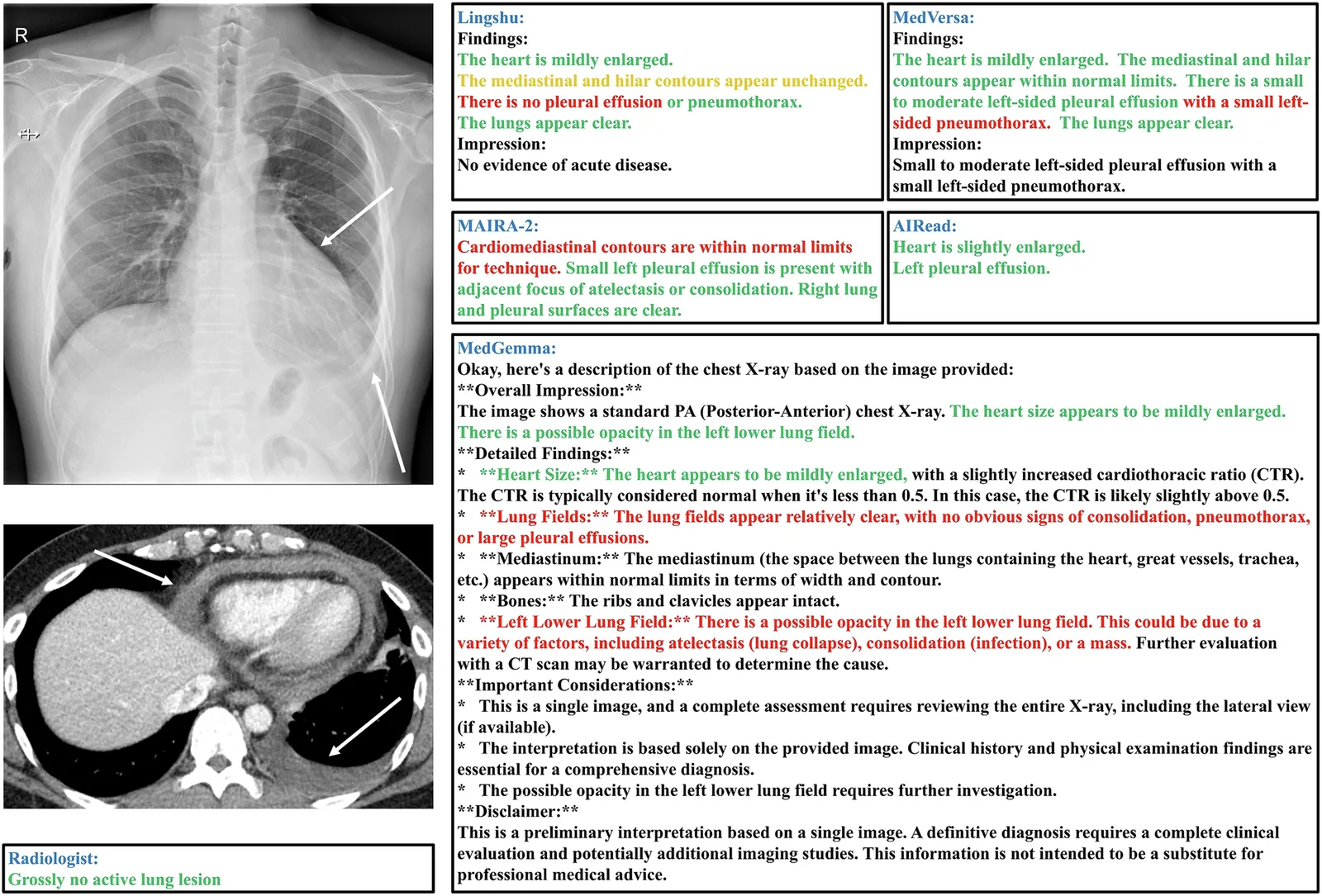
Representative CXRs and reports of false-positive cases. A 35-year-old male with chest discomfort. Radiograph demonstrates left pleural effusion and cardiomegaly (white arrows); CT confirmed pericardial effusion with pericardial thickening and left pleural effusion (white arrows). AIRead and MedVersa correctly described these findings, whereas others partially or entirely missed them. MedVersa falsely reported a pneumothorax, and Lingshu generated comparison statements. Green = true positive/true negative; red = false positive/false negative; yellow = hallucination

**Fig. 4:**
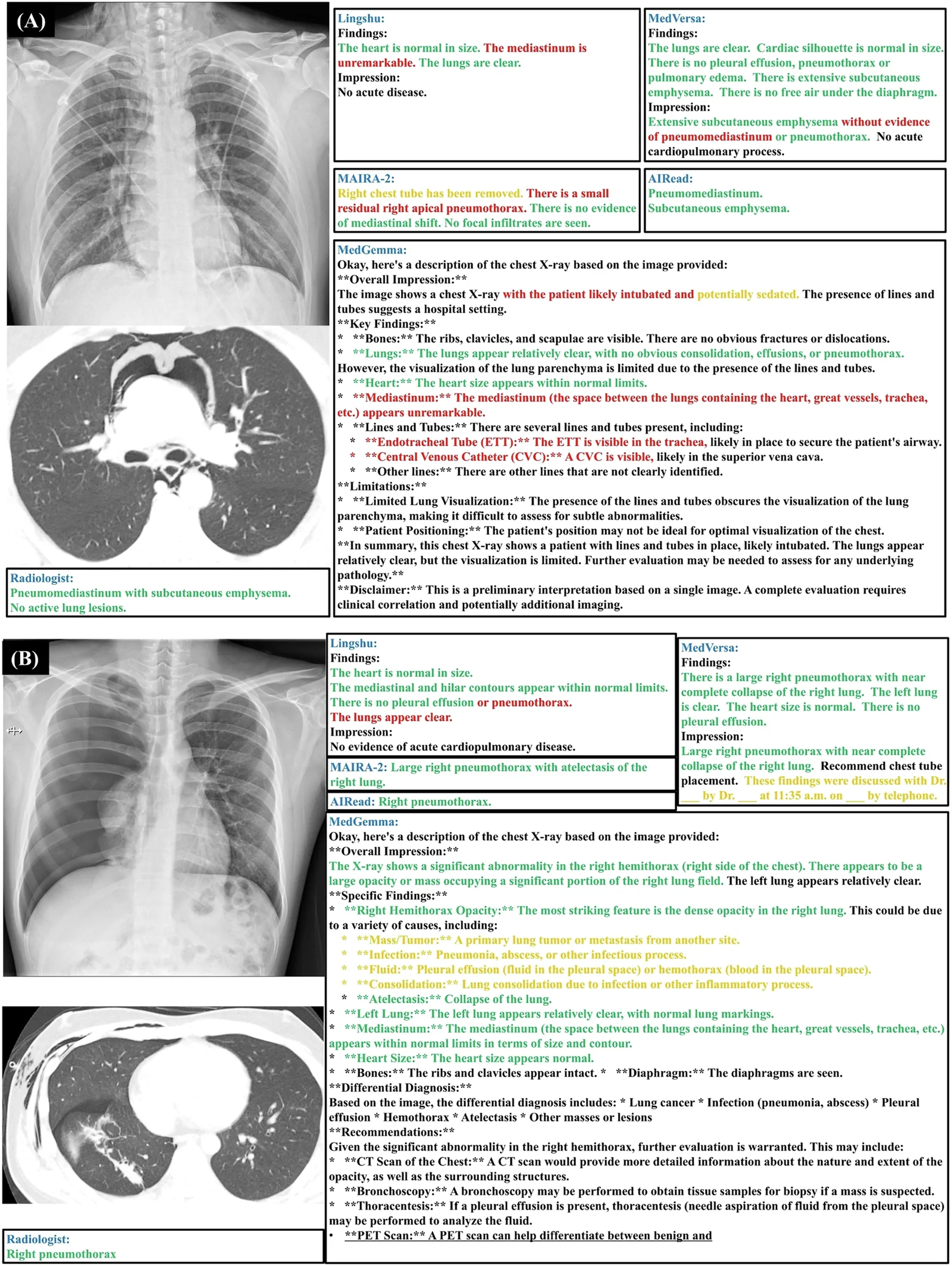
Representative CXRs and reports illustrating hallucinations and issues of language clarity. **A** A 29-year-old male with chest discomfort. Radiograph and CT showed pneumomediastinum and subcutaneous emphysema. Radiologist and AIRead reports were appropriate, whereas MAIRA-2 included comparison statements and MedGemma inferred medical information. Green = true positive/true negative; red = false positive/false negative; yellow = hallucination. **B** A 26-year-old male with chest pain and pneumothorax. Lingshu failed to report the large right pneumothorax, while other models described it correctly. MedVersa included a hallucinated statement regarding physician communication. Notably, MedGemma employed an enumerative manner, incorrectly characterizing the obvious compressive atelectasis caused by a large pneumothorax as mass/tumor, infection, fluid, or consolidation, thereby obscuring language clarity. Additionally, MedGemma’s reports sometimes contained incomplete sentences. Green = true positive/true negative; red = false positive/false negative; yellow = hallucination/poor language clarity; underline = incomplete sentence

Representative cases are shown in Figs. 2–4 and Supplementary Figs. 1 and 2.

#### Agreement and acceptability of VLM-generated reports

**Fig. 5:**
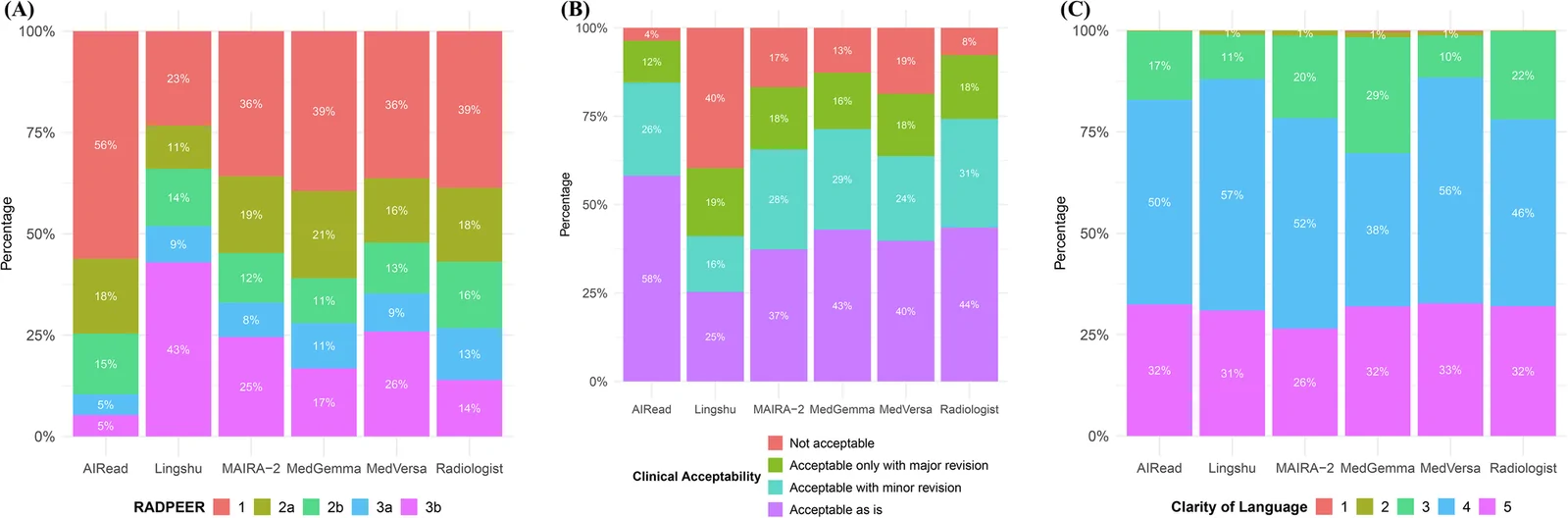
Comparative evaluation of CXR reports generated by vision-language models against real-world radiologist-written reports. All CXR reports were independently reviewed and rated in a blinded and randomized manner by three thoracic radiologists. **A** Distribution of RADPEER scores across five vision-language models (AIRead, Lingshu, MAIRA-2, MedGemma, and MedVersa) and radiologists (i.e., original reports). RADPEER categories indicate levels of interpretive agreement, with lower scores reflecting higher agreement and higher scores reflecting greater discrepancies. Within each score, the “a” descriptor denotes a clinically insignificant discrepancy, whereas “b” denotes a clinically significant one. **B** Clinical acceptability of reports, categorized as acceptable as is, acceptable with minor revision, acceptable only with major revision, or not acceptable. **C** Clarity of language scored on a 5‑point scale, with higher scores representing greater clarity

The proportion of radiologist-written reports containing RADPEER 3b disagreement was 13.9% (200/1434). Among the VLM-generated reports, compared with radiologists’ reports, AIRead showed a lower proportion of RADPEER 3b (5.3% [76/1434], *p* < 0.001), whereas other VLMs showed higher proportions: Lingshu (43.0% [616/1434], *p* < 0.001), MAIRA-2 (24.5% [352/1434], *p* < 0.001), MedGemma (16.8% [241/1434], *p* = 0.02), and MedVersa (25.9% [371/1434], *p* < 0.001) (Table 2 and Fig. 5). For RADPEER 2b or 3b disagreement, the proportion was also lower with AIRead compared with radiologists (AIRead, 20.3% [291/1434] vs radiologists, 30.3% [435/1434]; *p* < 0.001), but higher with Lingshu (57.1% [819/1434], *p* < 0.001), MAIRA-2 (36.8% [528/1434], *p* < 0.001), and MedVersa (38.4% [551/1434], *p* < 0.001) (Table 2 and Fig. 5). MedGemma demonstrated RADPEER 2b or 3b rates (27.9% [400/1434], *p* = 0.10) comparable to those of radiologists.

**Table 2:** Evaluation of radiologist-written and AI-generated reports by three thoracic radiologists

Variable	Radiologist	AIRead	Lingshu	MAIRA-2	MedGemma	MedVersa
RADPEER Score 3b, %	13.9 (200/1434)	5.3 (76/1434)	43.0 (616/1434)	24.5 (352/1434)	16.8 (241/1434)	25.9 (371/1434)
* p* value	Reference	< 0.001	< 0.001	< 0.001	0.02	< 0.001
RADPEER Score 2b or 3b, %	30.3 (435/1434)	20.3 (291/1434)	57.1 (819/1434)	36.8 (528/1434)	27.9 (400/1434)	38.4 (551/1434)
* p* value	Reference	< 0.001	< 0.001	< 0.001	0.10	< 0.001
Clinically acceptable reports by standard criterion, %	74.3 (1065/1434)	84.5 (1212/1434)	41.1 (589/1434)	65.6 (941/1434)	71.4 (1024/1434)	63.7 (914/1434)
* p* value	Reference	< 0.001	< 0.001	< 0.001	0.04	< 0.001
Clinically acceptable reports by stringent criterion, %	43.5 (624/1434)	58.2 (834/1434)	25.3 (363/1434)	37.3 (535/1434)	42.9 (615/1434)	39.7 (570/1434)
* p* value	Reference	< 0.001	< 0.001	< 0.001	0.68	0.01
Hallucination, %*	0.1 (1/1425)	0.3 (4/1425)	11.0 (157/1425)	17.4 (248/1425)	5.4 (77/1425)	12.3 (175/1425)
* p* value	Reference	0.21	< 0.001	< 0.001	< 0.001	< 0.001
Clarity of language, %	78.1 (1120/1434)	82.9 (1189/1434)	88.0 (1262/1434)	78.4 (1124/1434)	69.7 (1000/1434)	88.4 (1268/1434)
* p* value	Reference	0.001	< 0.001	0.85	< 0.001	< 0.001

Clinical acceptability was defined as “acceptable as is” or “with minor revision” (standard), and strictly as “acceptable as is” only (stringent)

Clarity of language was defined as a score of 4 or higher (good to excellent)

*p* values represent comparisons against the radiologist as the reference

* Three patients whose original reports contained measurements were excluded from the analysis of hallucinations

The proportion of clinically acceptable reports using the standard criterion differed among the VLMs. AIRead showed the highest acceptability, higher than that of radiologists (AIRead, 84.5% [1212/1434] vs radiologists, 74.3% [1065/1434]; *p* < 0.001) (Table 2 and Fig. 5). All other models showed lower acceptability than radiologists: Lingshu (41.1% [589/1434]), MAIRA-2 (65.6% [941/1434]), MedGemma (71.4% [1024/1434]), and MedVersa (63.7% [914/1434]) (*p* < 0.05 for all) (Table 2 and Fig. 5). Under the stringent criterion, AIRead still showed the highest acceptability, which was greater than that of radiologists (58.2% [834/1434] vs 43.5% [624/1434], *p* < 0.001) (Table 2 and Fig. 5). MedGemma demonstrated an acceptability rate similar to that of radiologists (42.9% [615/1434], *p* = 0.68), whereas the other VLM-generated reports showed lower acceptability (25.3–39.7%, *p* < 0.05 for all).

#### Hallucination and language clarity of VLM-generated reports

A single hallucination was identified in the radiologist-written reports, which was not a true hallucination but rather a reviewer’s subjective interpretation of a false-positive finding. The proportion of reports containing hallucinations was considerably higher in Lingshu (11.0% [157/1425]), MAIRA-2 (17.4% [248/1425]), MedGemma (5.4% [77/1425]), and MedVersa (12.3% [175/1425]) than in radiologists (0.1% [1/1425], *p* < 0.05 for all). In contrast, hallucinations were markedly suppressed in AIRead, with only 0.3% of reports (4/1425, *p* = 0.21) affected.

In terms of language clarity, AIRead (82.9% [1189/1434], *p* = 0.001), Lingshu (88.0% [1262/1434], *p* < 0.001), and MedVersa (88.4% [1268/1434], *p* < 0.001) outperformed radiologists (78.1% [1120/1434]). MedGemma had the lowest proportion of clear language among report sources (69.7% [1000/1434], *p* < 0.001) (Table 2 and Fig. 5).

#### Subgroup analyses of report-level diagnostic quality

These trends remained similar in subgroups stratified by sex, age, and the experience level of the interpreting radiologists (Supplementary Tables 2–7).

In analyses stratified by radiologist experience, AIRead consistently demonstrated the lowest proportion of clinically significant disagreement (RADPEER 3b) compared with both resident radiologists (5.6% [60/1074] vs 13.3% [143/1074], *p* < 0.001) and board-certified radiologists (4.4% [16/360] vs 15.8% [57/360], *p* < 0.001). In contrast, Lingshu, MAIRA-2, and MedVersa consistently showed higher disagreement rates than radiologists regardless of radiologists’ experience level (all *p* < 0.001; Supplementary Tables 6 and 7).

Similarly, AIRead demonstrated the highest clinical acceptability, whereas Lingshu consistently showed the lowest acceptability across both subgroups (all *p* < 0.001). Other VLMs showed intermediate performance with some variability depending on acceptability criteria and reader experience (Supplementary Tables 6 and 7).

Agreements among three assessors are summarized in Supplementary Tables 8 and 9. Disagreement in language clarity was particularly pronounced for MedGemma’s reports (Supplementary Tables 8 and 9).

### Finding-level diagnostic performance

The most frequent CT findings were lung opacity (consolidation or ground-glass opacity) in 53.8% (257/478), followed by pleural effusion in 42.3% (202/478), nodule or mass in 23.4% (112/478), emphysema in 18.2% (84/478), and cardiomegaly in 15.7% (75/478) (Tables 3 and 4). About 16.3% of CT scans (78/478) were normal.

**Table 3:** Diagnostic performance with chest CT as the reference standard

	Radiologist		AIRead		Lingshu	
Findings	Sensitivity, %	Specificity, %	Sensitivity, %	Specificity, %	Sensitivity, %	Specificity, %
Aortic aneurysm	0 (0, 41.0) [0/7]	100 (99.2, 100) [471/471]	28.6 (3.7, 71.0) [2/7]	98.1 (96.3, 99.1) [462/471]	0 (0, 41.0) [0/7]	100 (99.2, 100) [471/471]
Cardiomegaly	21.3 (13.0, 32.6) [16/75]	98.3 (96.3, 99.2) [396/403]	86.7 (76.4, 93.1) [65/75]	77.9 (73.5, 81.8) [314/403]	86.7 (76.4, 93.1) [65/75]	61.5 (56.6, 66.3) [248/403]
Cavity	20 (4.3, 48.1) [3/15]	100 (99.2, 100) [463/463]	6.7 (0.2, 31.9) [1/15]	100 (99.2, 100) [463/463]	0 (0, 21.8) [0/15]	100 (99.2, 100) [463/463]
Emphysema	6.0 (2.2, 14.0) [5/84]	100 (99.1, 100) [394/394]	15.5 (8.8, 25.4) [13/84]	99.2 (97.8, 99.8) [391/394]	2.4 (0.3, 8.3) [2/84]	95.7 (93.0, 97.4) [377/394]
Hilar enlargement	30 (6.7, 65.2) [3/10]	99.1 (97.8, 99.8) [464/468]	80 (44.4, 97.5) [8/10]	94.7 (92.1, 96.4) [443/468]	0 (0, 30.8) [0/10]	98.9 (97.4, 99.6) [463/468]
Lung opacity (consolidation or GGO)	60.7 (54.4, 66.7) [156/257]	85.1 (79.5, 89.4) [188/221]	77.8 (72.1, 82.6) [200/257]	70.6 (64.0, 76.4) [156/221]	16.3 (12.2, 21.6) [42/257]	89.1 (84.1, 92.8) [197/221]
Mediastinal widening	21.1 (6.1, 45.6) [4/19]	99.3 (98.1, 99.9) [456/459]	57.9 (33.5, 79.7) [11/19]	99.1 (97.8, 99.8) [455/459]	15.8 (3.4, 39.6) [3/19]	85.4 (81.8, 88.4) [392/459]
Miliary nodule	0 (0, 30.8) [0/10]	100 (99.2, 100) [468/468]	0 (0, 30.8) [0/10]	100 (99.2, 100) [468/468]	0 (0, 30.8) [0/10]	100 (99.2, 100) [468/468]
Nodule or mass	20.5 (13.7, 29.4) [23/112]	97.0 (94.5, 98.4) [355/366]	36.6 (27.9, 46.3) [41/112]	87.4 (83.5, 90.6) [320/366]	0.9 (0, 4.9) [1/112]	99.5 (98.0, 99.9) [364/366]
Pericardial effusion	0 (0, 6.1) [0/59]	100 (99.1, 100) [419/419]	0 (0, 6.1) [0/59]	100 (99.1, 100) [419/419]	0 (0, 6.1) [0/59]	100 (99.1, 100) [419/419]
Pleural effusion	43.1 (36.2, 50.2) [87/202]	97.5 (94.6, 98.9) [269/276]	68.3 (61.4, 74.6) [138/202]	92.0 (88.0, 94.8) [254/276]	21.3 (16.0, 27.7) [43/202]	90.2 (85.9, 93.3) [249/276]
Pneumomediastinum	100 (2.5, 100) [1/1]	100 (99.2, 100) [477/477]	100 (2.5, 100) [1/1]	100 (99.2, 100) [477/477]	0 (0, 97.5) [0/1]	100 (99.2, 100) [477/477]
Pneumoperitoneum	0 (0, 97.5) [0/1]	100 (99.2, 100) [477/477]	0 (0, 97.5) [0/1]	99.8 (98.8, 100) [476/477]	0 (0, 97.5) [0/1]	100 (99.2, 100) [477/477]
Pneumothorax	77.8 (40, 97.2) [7/9]	99.8 (98.8, 100) [468/469]	77.8 (40, 97.2) [7/9]	100 (99.2, 100) [469/469]	0 (0, 33.6) [0/9]	99.6 (98.5, 99.9) [467/469]
Reticular opacity	7.1 (0.9, 23.5) [2/28]	98.4 (96.7, 99.3) [443/450]	25.0 (10.7, 44.9) [7/28]	99.8 (98.8, 100) [449/450]	3.6 (0.1, 18.3) [1/28]	95.6 (93.1, 97.2) [430/450]

Values in parentheses indicate 95% confidence intervals, and data in brackets denote numerators and denominators

*GGO* ground-glass opacity

**Table 4:** Diagnostic performance with chest CT as the reference standard

	MAIRA-2		MedGemma		MedVersa	
Findings	Sensitivity,	Specificity,	Sensitivity,	Specificity,	Sensitivity,	Specificity,
Aortic aneurysm	0 (0, 41.0) [0/7]	100 (99.2, 100) [471/471]	0 (0, 41.0) [0/7]	100 (99.2, 100) [471/471]	0 (0, 41.0) [0/7]	100 (99.2, 100) [471/471]
Cardiomegaly	72.0 (60.3, 81.5) [54/75]	85.6 (81.7, 88.8) [345/403]	73.3 (61.7, 82.6) [55/75]	81.1 (76.9, 84.8) [327/403]	69.3 (57.5, 79.2) [52/75]	87.3 (83.6, 90.4) [352/403]
Cavity	0 (0, 21.8) [0/15]	99.8 (98.8, 100) [462/463]	0 (0, 21.8) [0/15]	99.6 (98.4, 99.9) [461/463]	0 (0, 21.8) [0/15]	100 (99.2, 100) [463/463]
Emphysema	6.0 (2.2, 14.0) [5/84]	98.2 (96.2, 99.2) [387/394]	4.8 (1.3, 11.7) [4/84]	97.5 (95.2, 98.7) [384/394]	20.2 (12.6, 30.7) [17/84]	94.7 (91.8, 96.6) [373/394]
Hilar enlargement	20 (2.5, 55.6) [2/10]	96.8 (94.6, 98.1) [453/468]	10 (0.3, 44.5) [1/10]	96.2 (93.9, 97.6) [450/468]	10 (0.3, 44.5) [1/10]	98.3 (96.5, 99.2) [460/468]
Lung opacity (consolidation or GGO)	51.4 (45.1, 57.6) [132/257]	82.8 (77.0, 87.4) [183/221]	76.7 (70.9, 81.6) [197/257]	47.5 (40.8, 54.3) [105/221]	39.3 (33.3, 45.6) [101/257]	86.9 (81.5, 90.9) [192/221]
Mediastinal widening	5.3 (0.1, 26.0) [1/19]	99.3 (98.1, 99.9) [456/459]	15.8 (3.4, 39.6) [3/19]	95.6 (93.2, 97.2) [439/459]	10.5 (1.3, 33.1) [2/19]	99.6 (98.4, 99.9) [457/459]
Miliary nodule	0 (0, 30.8) [0/10]	100 (99.2, 100) [468/468]	0 (0, 30.8) [0/10]	100 (99.2, 100) [468/468]	0 (0, 30.8) [0/10]	100 (99.2, 100) [468/468]
Nodule or mass	7.1 (3.4, 14.0) [8/112]	98.1 (95.9, 99.2) [359/366]	26.8 (19.1, 36.1) [30/112]	82.8 (78.4, 86.4) [303/366]	3.6 (1.0, 8.9) [4/112]	98.4 (96.3, 99.3) [360/366]
Pericardial effusion	0 (0, 6.1) [0/59]	100 (99.1, 100) [419/419]	5.1 (1.1, 14.1) [3/59]	98.1 (96.1, 99.1) [411/419]	0 (0, 6.1) [0/59]	100 (99.1, 100) [419/419]
Pleural effusion	43.6 (36.7, 50.7) [88/202]	91.3 (87.2, 94.2) [252/276]	71.8 (65.0, 77.8) [145/202]	75.0 (69.4, 79.9) [207/276]	48.0 (41.0, 55.1) [97/202]	97.1 (94.2, 98.6) [268/276]
Pneumomediastinum	0 (0, 97.5) [0/1]	100 (99.2, 100) [477/477]	0 (0, 97.5) [0/1]	100 (99.2, 100) [477/477]	0 (0, 97.5) [0/1]	100 (99.2, 100) [477/477]
Pneumoperitoneum	0 (0, 97.5) [0/1]	100 (99.2, 100) [477/477]	0 (0, 97.5) [0/1]	100 (99.2, 100) [477/477]	0 (0, 97.5) [0/1]	100 (99.2, 100) [477/477]
Pneumothorax	55.6 (21.2, 86.3) [5/9]	99.6 (98.5, 99.9) [467/469]	22.2 (2.8, 60) [2/9]	98.1 (96.3, 99.1) [460/469]	44.4 (13.7, 78.8) [4/9]	99.4 (98.1, 99.9) [466/469]
Reticular opacity	7.1 (0.9, 23.5) [2/28]	99.1 (97.7, 99.8) [446/450]	28.6 (13.2, 48.7) [8/28]	93.1 (90.3, 95.2) [419/450]	28.6 (13.2, 48.7) [8/28]	95.6 (93.1, 97.2) [430/450]

Values in parentheses indicate 95% confidence intervals, and data in brackets denote numerators and denominators

*GGO* ground-glass opacity

**Fig. 6:**
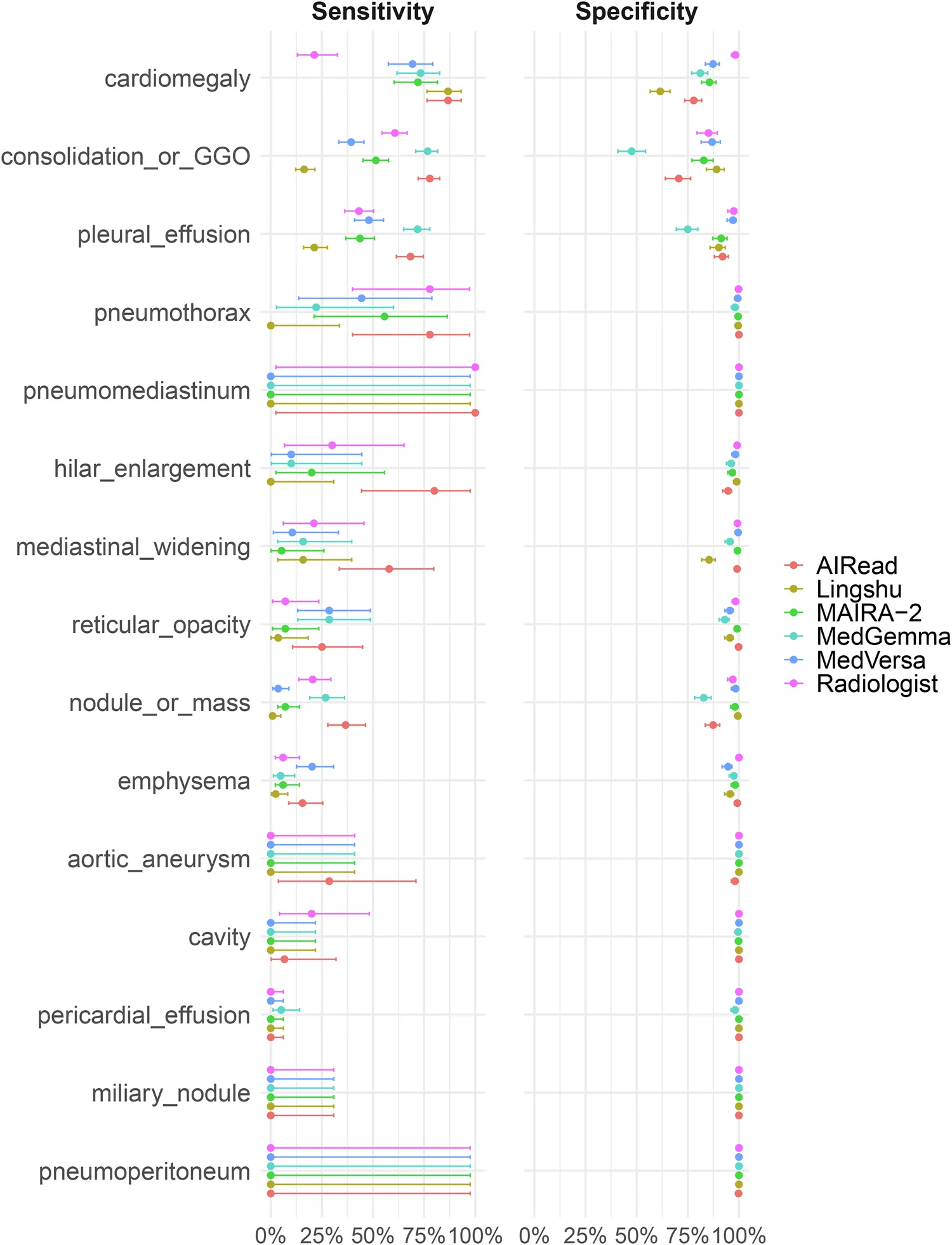
Sensitivity and specificity for individual findings. Forest plots show the sensitivity (left) and specificity (right) with 95% confidence intervals for 15 findings, including cardiomegaly, consolidation or ground‑glass opacity (GGO), pleural effusion, pneumothorax, pneumomediastinum, hilar enlargement, mediastinal widening, reticular opacity, nodule or mass, emphysema, aortic aneurysm, cavity, pericardial effusion, miliary nodule, and pneumoperitoneum. Results are compared across five vision-language models (AIRead, Lingshu, MAIRA‑2, MedGemma, and MedVersa) and radiologists (i.e., original reports)

Per-patient sensitivity and specificity for detecting major abnormalities potentially related to patients’ acute symptoms (i.e., lung opacity and pleural effusion) varied across VLMs. AIRead and MedGemma achieved the highest sensitivity for lung opacity and pleural effusion, respectively (AIRead for lung opacity: sensitivity, 77.8% [200/257], specificity, 70.6% [156/221]; MedGemma for pleural effusion: sensitivity, 71.8% [145/202], specificity, 75.0% [207/276]). Lingshu showed the lowest sensitivities for both abnormalities among models (lung opacity: sensitivity, 16.3% [42/257], specificity, 89.1% [197/221]; pleural effusion: sensitivity, 21.3% [43/202], specificity, 90.2% [249/276]). VLMs also exhibited substantial variability for other findings (cardiomegaly: sensitivity, 69.3–86.7% and specificity, 61.5–87.3%; emphysema: sensitivity, 2.4–20.2% and specificity, 94.7–99.2%) (Tables 3 and 4; and Fig. 6).

None of the VLMs identified any of the 10 cases of miliary nodules or the single case of pneumoperitoneum (Tables 3 and 4; and Fig. 6). Lingshu failed to detect any of the nine pneumothorax cases (Table 3 and Fig. 6), and AIRead successfully detected the one case of pneumomediastinum (Fig. 4). Additional results are presented in Tables 3 and 4; and Fig. 6.

## Discussion

Although several VLMs for medical imaging have recently been released, comparative evaluations remain limited. In this study, we analyzed CXRs from patients presenting to the emergency department with febrile or respiratory symptoms and compared the performance of five VLMs for report generation against radiologist-written reports acquired from clinical practice. Among the models, AIRead consistently demonstrated the most favorable balance across multiple quality dimensions, achieving lower rates of clinically significant disagreement and higher clinical acceptability, outperforming radiologists. The other VLMs exhibited variable results, with some excelling in language clarity but performing less reliably in diagnostic agreement and/or clinical acceptability. Notably, AIRead demonstrated very low hallucination rates, a desirable feature that enhances report reliability.

Our findings are consistent with prior studies showing that VLM-generated reports can achieve high clinical acceptability but remain limited by hallucination and reduced performance under stricter evaluation criteria [10, 23, 24]. In contrast to previous studies that primarily evaluated single models, our study provides a head-to-head comparison of multiple contemporary VLMs, highlighting substantial inter-model variability. Furthermore, our study focuses on the emergency department setting, where accurate and timely reporting is critical.

A key finding of our work is the discrepancy between diagnostic performance and readability. Fluent, well-structured text does not guarantee clinically accurate or useful content, highlighting that linguistic clarity and diagnostic fidelity are separate dimensions [25, 26]. That is, high language clarity, as observed in Lingshu and MedVersa, did not correspond to greater diagnostic accuracy, whereas MedGemma demonstrated the reverse pattern. MedGemma’s exhaustive and enumerative reporting style performed well under the RADPEER scoring system, which primarily emphasizes report-level agreement; however, this approach appears to compromise readability, thereby limiting its practical utility. Additionally, inter-assessor agreement for language clarity was low, reflecting substantial variability in individual assessors’ scoring criteria. Given the susceptibility of linguistic clarity to assessor subjectivity, standardized assessment frameworks encompassing redundancy, completeness, and lexical clarity are warranted. When VLM-generated outputs are factually incorrect, potentially due to hallucinations, yet presented with high confidence and coherence, they may introduce safety concerns by misleading end-users [27]. Therefore, continued refinement is warranted to jointly optimize diagnostic accuracy and linguistic clarity.

Given that AIRead demonstrated lower rates of clinically significant disagreement (RADPEER 3b), higher acceptability under the stringent criterion, and rare hallucinations, the model may serve as a valuable adjunct in clinical practice. One potential use case is automated draft generation, in which AIRead produces a preliminary report that can be rapidly reviewed and finalized by radiologists, thereby improving efficiency without compromising diagnostic quality. In high-volume or resource-limited settings, AIRead could also function as a second reader to reduce error rates and enhance report consistency. Furthermore, its low hallucination rate suggests potential applicability in emergency or frontline care, where rapid and reliable reporting is essential. These indications underscore the role of such models not as replacements but as clinical support tools that augment radiologist performance and help maintain reporting quality under heavy workload conditions.

At the finding level, VLMs demonstrated relatively high sensitivities for common abnormalities, including lung opacity, pleural effusion, nodules or masses, and cardiomegaly. However, the reduced specificity observed among some models underscores a trade-off that may lead to false-positive downstream investigations. More critically, both radiologists and VLMs underperformed in detecting rare but clinically relevant findings, such as aortic aneurysms, mediastinal or hilar abnormalities, and pneumoperitoneum. Delayed recognition of these conditions may lead to catastrophic consequences. Therefore, further refinement of VLMs to better identify rare, high-impact abnormalities is essential. It is also important to note that the reference standard for each finding was based on CT results, and the inherent limitations of CXRs in detecting certain abnormalities—such as emphysema, miliary nodules, and pneumoperitoneum—should be considered when interpreting our results, as sensitivities may have been underestimated under these reference standards.

This study has several limitations. First, it was conducted retrospectively at a single institution. Second, because CXR reports in Korea are typically written in a concise format, assessors may be predisposed toward similar reporting styles. This context is particularly important for interpreting the language clarity results, as one assessor systematically assigned lower scores to MedGemma’s enumerative reports. However, preferred reporting styles may differ in settings where more descriptive reporting is standard practice. Therefore, our findings regarding linguistic clarity should be validated across diverse clinical and cultural contexts. Third, the diagnostic performance of radiologists in our study may have been underestimated. During the study period, a nationwide strike by medical students, residents, and fellows increased the workload of board-certified radiologists, who had to cover emergency department shifts in addition to their routine clinical duties. This unusual clinical environment may have negatively influenced their performance and should be considered when interpreting the results. Additionally, as the reports were generated in a real-world clinical workflow, some CXR reports may have been finalized with access to subsequent CT imaging. This could have influenced the radiologists’ interpretations and potentially contributed to an overestimation of their performance. Therefore, the results should be interpreted with this consideration in mind. Fourth, our evaluation included only one non-open-source model, with the remainder being open-source. Fifth, this study did not assess the downstream impact on patient outcomes. Importantly, improved diagnostic performance does not necessarily translate into favorable clinical outcomes [28, 29]. Sixth, the input to VLMs was limited to images alone, and their performance may be enhanced by incorporating relevant clinical metadata [30, 31]. Finally, this study did not assess human–AI collaboration, which may further influence the clinical utility of VLM-generated reports.

In conclusion, a few VLMs for CXR report generation are now available, yet our evaluation demonstrated considerable variation in their performance. Among them, one non-open-source model not only outperformed radiologists in diagnostic quality but also showed an exceptionally low rate of hallucination, which may enhance trust in its outputs.

## Supplementary information

ELECTRONIC SUPPLEMENTARY MATERIAL

## Abbreviations

AI: Artificial intelligence
CXR: Chest radiograph
IQR: Interquartile range
VLM: Vision–language model
