# Supplementary material for: Comparative evaluation of generative AI models for chest radiograph report generation in the emergency department
Source: Eur Radiol. 2026 Jun 10;36(10):7911–24. doi: 10.1007/s00330-026-12648-8 (PMC13574732; doi:10.1007/s00330-026-12648-8)
Supplement: Supplementary file 1 [file 330_2026_12648_MOESM1_ESM.pdf]

Comparative Evaluation of Generative AI Models for Chest Radiograph Report Generation in the Emergency Department

ELECTRONIC SUPPLEMENTARY MATERIALS

Supplementary Texts

Supplementary Text 1: Medical image–specific vision–language models

(1) AIRead: AIRead was designed to generate reports from frontal chest radiographs (CXRs) (either anteroposterior or posteroanterior view). The model comprises an image encoder and a language model; the encoder localizes potential abnormal regions and assigns 61 finding categories, and these outputs are passed to the language model for report generation. The model contains 2.6 billion parameters. The performance of an early version has been reported previously [1, 2]. The initial training dataset comprised approximately 14 million CXRs paired with reports, retrospectively collected from 11 tertiary hospitals in Korea and from multiple hospitals and clinics in the United States, of which 8 million image–report pairs were used to train the image encoder. Unlike the other models used in our experiments, the parameters of AIRead are not publicly available. However, the model can be accessed at https://airead.soombit.ai/, and the version used in this study was v0.2.5. This model is for research use only and has not been cleared or approved by regulatory authorities for clinical use.

(2) Lingshu: Lingshu emphasizes comprehensive data curation to enhance medical artificial intelligence reasoning across multiple tasks, including multimodal question answering, text-based question answering, and medical report generation [3]. The model accepts diverse imaging modalities such as plain radiograph, CT, MRI, ultrasound, dermoscopy, fundus photography, histopathology, and microscopy. The training dataset comprised 2.55 million instances, with x-ray images accounting for 13%. Lingshu was built on the Qwen2.5-VL-Instruct model, with 7 billion and 32 billion parameters. In a pilot evaluation with a thoracic radiologist, the 32B model performed comparably to the 7B model for CXR report generation. Therefore, for these experiments, we used the 7B parameters available at https://huggingface.co/lingshu-medical-mllm/Lingshu-7B and followed the inference recommendations provided in the model card.

(3) MAIRA-2: MAIRA-2 was developed to provide comprehensive support for radiologists in interpreting CXRs by leveraging both frontal and lateral views, prior studies, and clinical indication [4].

The model further enhances report interpretation through a novel task termed grounded report generation, which explicitly localizes each finding within the image. MAIRA-2 consists of an image encoder and a large language model with a visual adapter, using the radiology-specialized Rad-DINO-MAIRA-2 as the image encoder and Vicuna 7B v1.5 as the language model. The total model size is approximately 6.9 billion parameters. The training dataset included 510K image–report pairs from MIMIC-CXR, PadChest, USMix, and IU-Xray. For these experiments, we used the model parameters available at https://huggingface.co/microsoft/maira-2. For inference, we used "without grounding option", since the quality of the report only differs slightly as described in the model card.

(4) MedGemma: MedGemma is a medical foundation model designed to perform across diverse clinical tasks while reducing task-specific tuning requirements, thereby accelerating the development of artificial intelligence for healthcare applications [5]. Its vision module processes four imaging domains: radiology (CXRs, CT, MRI), histopathology, dermatology, and ophthalmology. Supported tasks include medical visual question answering, medical image classification, CXR report generation, and related applications. The architecture comprises a visual encoder and a language model connected via a visual adapter; the visual encoder is fine-tuned from SigLIP, and the language model is based on Gemma 3. Like MedVersa, MIMIC-CXR was the primary training dataset for CXR report generation. MedGemma is released in multiple variants, including instruction-tuned and pre-trained models, with parameter sizes ranging from 4 billion to 27 billion. At the start of this study, the 27B multimodal model was not available; therefore, we used the 4B instruction-tuned model (https://huggingface.co/google/medgemma-4b-it) and followed the inference recommendations provided in the model card.

(5) MedVersa: MedVersa is a medical artificial intelligence generalist developed for diverse tasks in medical image interpretation [6]. It was trained on 13 million annotated instances spanning 11 tasks across 3 imaging modalities: CXRs, CT scans, and dermoscopic images. Supported tasks include report generation, open-ended visual question answering, and related applications. For CXR report generation, the primary training dataset was MIMIC-CXR. After filtering, MedVersa utilized approximately 122K

CXRs paired with reports. The model consists of a 2D/3D visual encoder and a large language model, with an adapter employed to transfer visual information to the language model. The total model size is approximately 6.9 billion parameters. For these experiments, we used the model parameters available at https://huggingface.co/hyzhou/MedVersa_Internal. For CXR report generation, we followed the recommendations provided in the model card, including the specified environment setup.

(6) Inference settings: All inferences were performed on a single NVIDIA H100 graphics processing unit with CUDA 12.4. To minimize the effects of stochastic variation in the generation process, greedy decoding was used to generate reports across all methods. Because the evaluation of this study focused on reports from a single frontal CXR, models capable of incorporating prior reports, additional images (including lateral views), or clinical information (e.g., age) were instead provided with blank or no inputs for these fields.

(7) Prompts used for each model:

(A) Lingshu: "You are a helpful assistant. Please generate a report for the given images, including both findings and impressions. Return the report in the following format: Findings: {} Impression: {}."

(B) MedGemma: "Describe this X-ray"

(C) MedVersa: "Can you provide a radiology report for <img0>?"

(D) MAIRA-2: No prompt was required.

(E) AIRead-CXR: No prompt was required.

Supplementary Text 2: Details of a large language model–based labeler for extracting abnormal findings from free-text chest radiograph reports

A practical approach to quantitatively assess report-generation models is to compare labels extracted from model-generated and radiologist-written free-text reports. To this end, model-based labelers such as RadGraph and CheXpert++ have been developed to extract predefined labels and relations from radiology reports [7, 8]. More recently, leveraging the medical knowledge embedded in large language models (LLMs) has emerged as a promising direction for label extraction. In this line of work, CheXGPT utilized LLM-based medical knowledge to extract 13 labels (atelectasis, consolidation, effusion, fracture, hyperinflation, lung opacity, nodule, pleural lesion, pneumothorax, pulmonary edema, subcutaneous emphysema, subdiaphragmatic gas, widened mediastinal silhouette) [9]. CheXGPT also distilled the knowledge of a LLM into a lightweight language model, thereby enabling efficient label extraction and supporting a wide range of clinical applications.

For this study, we developed a labeler similar to CheXGPT by leveraging LLMs to extract labels, but the coverage extended from 13 to 76 abnormal categories. We adopted a similar training strategy, in which pseudo-label sets were first extracted from chest radiograph reports by using a LLM. These pseudo-labels were refined through domain-specific post-processing and subsequently used to supervise the training of a Bidirectional Encoder Representations from Transformers-based extraction model [10].

Supplementary References

1. Hong EK, Ham J, Roh B, et al (2025) Diagnostic Accuracy and Clinical Value of a Domain-specific Multimodal Generative AI Model for Chest Radiograph Report Generation. Radiology 314:e241476. https://doi.org/10.1148/radiol.241476.

2. Hong EK, Roh B, Park B, et al (2025) Value of Using a Generative AI Model in Chest Radiography Reporting: A Reader Study. Radiology 314:e241646. https://doi.org/10.1148/radiol.241646.

3. Xu W, Chan HP, Li L, et al (2025) Lingshu: A Generalist Foundation Model for Unified Multimodal Medical Understanding and Reasoning. Preprint at arXiv. https://doi.org/10.48550/arXiv.2506.07044. Posted June 8, 2025. Accessed April 21, 2026.

4. Bannur S, Bouzid K, Castro DC, et al (2024) MAIRA-2: Grounded Radiology Report Generation. Preprint at arXiv. https://doi.org/10.48550/arXiv.2406.04449. Posted June 6, 2024. Accessed April 21, 2026.

5. Sellergren A, Kazemzadeh S, Jaroensri T, et al (2025) MedGemma Technical Report. Preprint at arXiv. https://doi.org/10.48550/arXiv.2507.05201. Posted July 7, 2025. Accessed April 21, 2026.

6. Zhou HY, Acosta JN, Adithan S, Datta S, Topol EJ, Rajpurkar P (2024) MedVersa: A Generalist Foundation Model for Medical Image Interpretation. Preprint at arXiv. https://doi.org/10.48550/arXiv.2405.07988. Posted May 13, 2024. Accessed April 21, 2026.

7. Jain S, Agrawal A, Saporta A, et al (2021) RadGraph: Extracting Clinical Entities and Relations from Radiology Reports. Preprint at arXiv. https://doi.org/10.48550/arXiv.2106.14463. Posted June 28, 2021. Accessed April 21, 2026.

8. McDermott MBA, Hsu TMH, Weng WH, Ghassemi M, Szolovits P (2026) CheXpert++: Approximating the CheXpert labeler for Speed, Differentiability, and Probabilistic Output. Preprint at arXiv. https://doi.org/10.48550/arXiv.2006.15229. Posted June 26, 2020. Accessed April 21, 2026.

9. Gu J, You K, Cho HC, Kim J, Hong EK, Roh B (2024) CheX-GPT: Harnessing Large

Language Models for Enhanced Chest X-ray Report Labeling. Preprint at arXiv. https://doi.org/10.48550/arXiv.2401.11505. Posted January 21, 2024. Accessed April 21, 2026.

10. Devlin J, Chang MW, Lee K, Toutanova K (2018) BERT: Pre-training of Deep Bidirectional Transformers for Language Understanding. Preprint at arXiv. https://doi.org/10.48550/arXiv.1810.04805. Posted October 11, 2018. Accessed April 21, 2026.

Supplementary Tables

Supplementary Table 1: Summarization of Evaluation Metrics

**Criteria**	**Details**
**RADPEER score**	
**1**	Agree with interpretations.
**2**	Disagree with interpretations which are understandable misses.
	**a:** unlikely to be clinically significant.
	**b**: likely to be clinically significant.
**3**	Disagree with interpretations which are unacceptable misses.
	**a:** unlikely to be clinically significant.
	**b:** likely to be clinically significant.
**Clinical acceptability**	
**1: Not acceptable**	The report contains major factual errors (e.g., missed referable findings, false-positive findings, incorrect laterality or diagnosis) that make it unsafe for clinical use. The content is misleading or unreliable and cannot be used even after editing.
**2: Acceptable only with major revision**	The report contains one or more significant problems (e.g., omission of a finding, vague or clinically misleading wording, or potentially harmful misinterpretation) that require extensive edits across multiple sentences or restructuring. A radiologist would need to spend meaningful time reviewing and rewriting the report before it could be used.
**3: Acceptable only with minor revision**	The report is generally accurate and includes key findings, but may have minor issues such as slightly

	awkward phrasing, limited detail (e.g., lacking severity), or subtle clarification needs. It can be used after quick edits without changing core content.
**4: Acceptable as is**	The report is accurate, clear, and clinically actionable as written. It requires no edits and could be signed and submitted directly.
**Hallucination**	The presence of information that could not be derived solely from a single frontal radiograph, in which information on scale was not available (e.g., references to a lateral view, clinical history, comparative statements, or size or distance descriptions).
**Language clarity**	Subjective scoring using a 5-point scale (1 = unacceptable, 2 = poor, 3 = moderate, 4 = good, 5 = excellent).

Supplementary Table 2: Subgroup Analysis in Male

Variable	Radiologist	AIRead	Lingshu	MAIRA-2	MedGemma	MedVersa
RADPEER Score 3b, %	13.4 (113/846)	5.6 (47/846)	42.9 (363/846)	24.2 (205/846)	17.1 (145/846)	24.7 (209/846)
P Value	Reference	<.001	<.001	<.001	.01	<.001
RADPEER Score 2b or 3b, %	27.8 (235/846)	18.8 (159/846)	55.6 (470/846)	34.5 (292/846)	27.5 (233/846)	35.2 (298/846)
P Value	Reference	<.001	<.001	<.001	.90	<.001
Clinically Acceptable Reports by Standard Criterion, %	76.4 (646/846)	85.2 (721/846)	42.3 (358/846)	67.5 (571/846)	71.7 (607/846)	67.1 (568/846)
P Value	Reference	<.001	<.001	<.001	.01	<.001
Clinically Acceptable Reports by Stringent Criterion, %	46.2 (391/846)	59.6 (504/846)	26.7 (226/846)	38.1 (322/846)	42.7 (361/846)	42.7 (361/846)
P Value	Reference	<.001	<.001	<.001	.07	.07
Hallucination, %	0.1 (1/846)	0.4 (3/846)	6.9 (58/846)	19.3 (163/846)	5.0 (42/846)	11.5 (97/846)
P Value	Reference	.33	<.001	<.001	<.001	<.001
Clarity of Language, %	76.6 (648/846)	82.9 (701/846)	89.2 (755/846)	77.9 (659/846)	69.4 (587/846)	87.9 (744/846)
P Value	Reference	.001	<.001	.52	.001	<.001

Clinical acceptability was defined as “acceptable as is” or “with minor revision” (standard), and strictly as “acceptable as is” only (stringent).

Clarity of language was defined as a score of 4 or higher (good to excellent).

P values represent comparisons against the radiologist as the reference.

Supplementary Table 3: Subgroup Analysis in Female

Variable	Radiologist	AIRead	Lingshu	MAIRA-2	MedGemma	MedVersa
RADPEER Score 3b, %	14.8 (87/588)	4.9 (29/588)	43.0 (253/588)	25.0 (147/588)	16.3 (96/588)	27.6 (162/588)
P Value	Reference	<.001	<.001	<.001	.41	<.001
RADPEER Score 2b or 3b, %	34.0 (200/588)	22.4 (132/588)	59.4 (349/588)	40.1 (236/588)	28.4 (167/588)	43.0 (253/588)
P Value	Reference	<.001	<.001	.01	.02	<.001
Clinically Acceptable Reports by Standard Criterion, %	71.3 (419/588)	83.5 (491/588)	39.3 (231/588)	62.9 (370/588)	70.9 (417/588)	58.8 (346/588)
P Value	Reference	<.001	<.001	<.001	.88	<.001
Clinically Acceptable Reports by Stringent Criterion, %	39.6 (233/588)	56.1 (330/588)	23.3 (137/588)	36.2 (213/588)	43.2 (254/588)	35.5 (209/588)
P Value	Reference	<.001	<.001	.15	.14	.08
Hallucination, %*	0 (0/579)	0.2 (1/579)	17.1 (99/579)	14.7 (85/579)	6.0 (35/579)	13.5 (78/579)
P Value	Reference	N/A	N/A	N/A	N/A	N/A
Clarity of Language, %	80.3 (472/588)	83.0 (488/588)	86.2 (507/588)	79.1 (465/588)	70.2 (413/588)	89.1 (524/588)
P Value	Reference	.22	.006	.61	<.001	<.001

Clinical acceptability was defined as “acceptable as is” or “with minor revision” (standard), and strictly as “acceptable as is” only (stringent).

Clarity of language was defined as a score of 4 or higher (good to excellent).

P values represent comparisons against the radiologist as the reference.

*Three patients whose original reports contained measurements were excluded from the analysis of hallucinations.

N/A = not available.

Supplementary Table 4: Subgroup Analysis of Patients Aged 65 Years and Older

Variable	Radiologist	AIRead	Lingshu	MAIRA-2	MedGemma	MedVersa
RADPEER Score 3b, %	17.7 (140/792)	5.7 (45/792)	47.5 (376/792)	30.7 (243/792)	19.2 (152/792)	29.9 (237/792)
P Value	Reference	<.001	<.001	<.001	.38	<.001
RADPEER Score 2b or 3b, %	37.4 (296/792)	21.8 (173/792)	63.5 (503/792)	42.4 (336/792)	29.9 (237/792)	43.2 (342/792)
P Value	Reference	<.001	<.001	.02	<.001	.008
Clinically Acceptable Reports by Standard Criterion, %	69.7 (552/792)	85.0 (673/792)	34.0 (269/792)	58.6 (464/792)	68.2 (540/792)	58.6 (464/792)
P Value	Reference	<.001	<.001	<.001	.45	<.001
Clinically Acceptable Reports by Stringent Criterion, %	29.8 (236/792)	50.6 (401/792)	14.8 (117/792)	26.3 (208/792)	33.7 (267/792)	29.9 (237/792)
P Value	Reference	<.001	<.001	.08	.06	.95
Hallucination, %*	0.1 (1/786)	0.5 (4/786)	12.5 (98/786)	21.0 (165/786)	7.6 (60/786)	12.8 (101/786)
P Value	Reference	.21	<.001	<.001	<.001	<.001
Clarity of Language, %	79.2 (627/792)	84.6 (670/792)	84.3 (668/792)	73.4 (581/792)	67.2 (532/792)	85.6 (678/792)
P Value	Reference	.004	.006	.005	<.001	.001

Clinical acceptability was defined as “acceptable as is” or “with minor revision” (standard), and strictly as “acceptable as is” only (stringent).

Clarity of language was defined as a score of 4 or higher (good to excellent).

P values represent comparisons against the radiologist as the reference.

*Two patients whose original reports contained measurements were excluded from the analysis of hallucinations.

Supplementary Table 5: Subgroup Analysis in Patients Younger Than 65 Years

Variable	Radiologist	AIRead	Lingshu	MAIRA-2	MedGemma	MedVersa
RADPEER Score 3b, %	9.3 (60/642)	4.8 (31/642)	37.4 (240/642)	17.0 (109/642)	13.9 (89/642)	20.9 (134/642)
P Value	Reference	<.001	<.001	<.001	.003	<.001
RADPEER Score 2b or 3b, %	21.7 (139/642)	18.4 (118/642)	49.2 (316/642)	29.9 (192/642)	25.4 (163/642)	32.6 (209/642)
P Value	Reference	.09	<.001	<.001	.06	<.001
Clinically Acceptable Reports by Standard Criterion, %	79.9 (513/642)	84.0 (539/642)	49.8 (320/642)	74.3 (477/642)	75.4 (484/642)	70.1 (450/642)
P Value	Reference	.03	<.001	.004	.02	<.001
Clinically Acceptable Reports by Stringent Criterion, %	60.4 (388/642)	67.4 (433/642)	38.3 (246/642)	50.9 (327/642)	54.2 (348/642)	51.9 (333/642)
P Value	Reference	.001	<.001	<.001	.004	<.001
Hallucination, %*	0 (0/639)	0 (0/639)	9.2 (59/639)	13.0 (83/639)	2.7 (17/639)	11.6 (74/639)
P Value	Reference	N/A	N/A	N/A	N/A	N/A
Clarity of Language, %	76.8 (493/642)	80.8 (519/642)	92.5 (594/642)	84.6 (543/642)	72.9 (468/642)	91.9 (590/642)
P Value	Reference	.07	<.001	<.001	.10	<.001

Clinical acceptability was defined as “acceptable as is” or “with minor revision” (standard), and strictly as “acceptable as is” only (stringent).

Clarity of language was defined as a score of 4 or higher (good to excellent).

P values represent comparisons against the radiologist as the reference.

*One patient whose original report included a measurement was excluded from the hallucination analysis.

N/A = not available.

Supplementary Table 6: Subgroup Analysis of Chest Radiograph Reports by Resident Radiologists

Variable	Radiologist	AIRead	Lingshu	MAIRA-2	MedGemma	MedVersa
RADPEER Score 3b, %	13.3 (143/1074)	5.6 (60/1074)	43.3 (465/1074)	24.0 (258/1074)	15.4 (165/1074)	25.3 (272/1074)
P Value	Reference	<.001	<.001	<.001	.12	<.001
RADPEER Score 2b or 3b, %	29.3 (315/1074)	20.9 (225/1074)	56.5 (607/1074)	36.1 (388/1074)	26.6 (286/1074)	37.7 (405/1074)
P Value	Reference	<.001	<.001	<.001	.11	<.001
Clinically Acceptable Reports by Standard Criterion, %	75.1 (807/1074)	84.4 (906/1074)	41.4 (445/1074)	65.9 (708/1074)	72.9 (783/1074)	64.5 (693/1074)
P Value	Reference	<.001	<.001	<.001	.16	<.001
Clinically Acceptable Reports by Stringent Criterion, %	45.3 (487/1074)	58.3 (626/1074)	26.0 (279/1074)	37.2 (400/1074)	44.8 (481/1074)	40.5 (435/1074)
P Value	Reference	<.001	<.001	<.001	.75	.005
Hallucination, %*	0.1 (1/1065)	0.3 (3/1065)	11.2 (119/1065)	16.8 (179/1065)	3.9 (42/1065)	12.1 (129/1065)
P Value	Reference	.34	<.001	<.001	<.001	<.001
Clarity of Language, %	77.8 (836/1074)	83.1 (892/1074)	88.5 (951/1074)	79.2 (851/1074)	69.8 (750/1074)	88.5 (951/1074)
P Value	Reference	.002	<.001	.42	<.001	<.001

Clinical acceptability was defined as “acceptable as is” or “with minor revision” (standard), and strictly as “acceptable as is” only (stringent).

Clarity of language was defined as a score of 4 or higher (good to excellent).

P values represent comparisons against the radiologist as the reference.

*Three patients whose original reports included a measurement were excluded from the hallucination analysis.

Supplementary Table 7: Subgroup Analysis of Chest Radiograph Reports by Board-Certified Radiologists

Variable	Radiologist	AIRead	Lingshu	MAIRA-2	MedGemma	MedVersa
RADPEER Score 3b, %	15.8 (57/360)	4.4 (16/360)	41.9 (151/360)	26.1 (94/360)	21.1 (76/360)	27.5 (99/360)
P Value	Reference	<.001	<.001	<.001	.04	<.001
RADPEER Score 2b or 3b, %	33.3 (120/360)	18.3 (66/360)	58.9 (212/360)	38.9 (140/360)	31.7 (114/360)	40.6 (146/360)
P Value	Reference	<.001	<.001	.07	.58	.02
Clinically Acceptable Reports by Standard Criterion, %	71.7 (258/360)	85.0 (306/360)	40.0 (144/360)	64.7 (233/360)	66.9 (241/360)	61.4 (221/360)
P Value	Reference	<.001	<.001	.02	.11	.001
Clinically Acceptable Reports by Stringent Criterion, %	38.1 (137/360)	57.8 (208/360)	23.3 (84/360)	37.5 (135/360)	37.2 (134/360)	37.5 (135/360)
P Value	Reference	<.001	<.001	.85	.78	.85
Hallucination, %	0 (0/360)	0.3 (1/360)	10.6 (38/360)	19.2 (69/360)	9.7 (35/360)	12.8 (46/360)
P Value	Reference	N/A	N/A	N/A	N/A	N/A
Clarity of Language, %	78.9 (284/360)	82.5 (297/360)	86.4 (311/360)	75.8 (273/360)	69.4 (250/360)	88.1 (317/360)
P Value	Reference	.21	.007	.32	.003	.001

Clinical acceptability was defined as “acceptable as is” or “with minor revision” (standard), and strictly as “acceptable as is” only (stringent).

Clarity of language was defined as a score of 4 or higher (good to excellent).

P values represent comparisons against the radiologist as the reference.

N/A = not available.

Supplementary Table 8: Results of Fleiss' κ among Three Assessors

Variable		Total	Radiologist	AIRead	Lingshu	MAIRA-2	MedGemma	MedVersa
RADPEER Score 3b	Fleiss' κ	0.389	0.210	0.069	0.471	0.379	0.222	0.349
	95% CI	0.368–0.410	0.158–0.262	0.017–0.121	0.419–0.522	0.329–0.431	0.170–0.274	0.297–0.401
RADPEER Score 2b or 3b	Fleiss' κ	0.384	0.330	0.211	0.490	0.397	0.248	0.334
	95% CI	0.362–0.405	0.278–0.382	0.159–0.263	0.439–0.542	0.346–0.449	0.196–0.299	0.282–0.386
Clinically Acceptable Reports by Standard Criterion	Fleiss' κ	0.415	0.292	0.206	0.548	0.397	0.272	0.372
	95% CI	0.394–0.437	0.240–0.344	0.154–0.258	0.496–0.599	0.345–0.449	0.221–0.324	0.321–0.424
Clinically Acceptable Reports by Stringent Criterion	Fleiss' κ	0.476	0.444	0.335	0.642	0.493	0.385	0.473
	95% CI	0.455–0.497	0.392–0.496	0.283–0.387	0.590–0.694	0.441–0.545	0.333–0.437	0.421–0.525
Hallucination	Fleiss' κ	0.671	0.749	-0.003	0.571	0.784	0.190	0.776
	95% CI	0.650–0.692	0.697–0.800	-0.055–0.049	0.519–0.623	0.732–0.836	0.139–0.242	0.724–0.827
Clarity of Language	Fleiss' κ	-0.097	-0.121	-0.132	0.055	-0.045	-0.338	-0.022
	95% CI	-0.118–-0.076	-0.173–-0.070	-0.184–-0.081	0.004–0.107	-0.097–0.006	-0.390–-0.286	-0.074–0.030

CI = confidence interval.

Supplementary Table 9: Results of Percentage Agreement among Three Assessors

Variable		Total	Radiologist	AIRead	Lingshu	MAIRA-2	MedGemma	MedVersa
RADPEER Score 3b	Percentage Agreement	69.0%	71.5%	86.0%	61.1%	65.5%	67.4%	62.6%
	95% CI	67.2–70.7%	67.6–75.5%	82.6–88.9%	56.7–65.5%	61.3–69.5%	63.2–71.3%	58.2–66.7%
RADPEER Score 2b or 3b	Percentage Agreement	57.8%	57.5%	61.7%	62.6%	57.9%	54.6%	52.7%
	95% CI	56.1–59.7%	53.1–61.9%	57.3–66.1%	58.2–66.9%	53.3–62.3%	50.2–59.0%	48.3–57.1%
Clinically Acceptable Reports by Standard Criterion	Percentage Agreement	61.1%	59.4%	68.8%	67.2%	59.2%	55.4%	56.5%
	95% CI	59.3–62.9%	55.2–63.6%	64.6–72.8%	63.0–71.3%	55.0–63.8%	50.8–59.8%	51.9–60.9%
Clinically Acceptable Reports by Stringent Criterion	Percentage Agreement	61.9%	59.0%	51.5%	79.7%	64.4%	54.8%	62.1%
	95% CI	60.2–63.7%	54.8–63.6%	46.9–56.1%	75.9–83.3%	60.0–69.0%	50.6–59.2%	57.7–66.5%
Hallucination	Percentage Agreement	92.9%	99.6%	99.2%	87.4%	90.6%	87.7%	92.7%
	95% CI	91.9–93.8%	99.0–100%	98.3–100%	84.3–90.2%	87.9–93.1%	84.3–90.6%	90.4–95.0%
Clarity of Language	Percentage Agreement	49.2%	42.5%	51.9%	70.1%	46.9%	15.3%	68.6%

	95% CI	47.4–50.9%	38.3–46.7%	47.5–56.1%	65.9–74.3%	42.3–51.5%	11.9–18.6%	64.6–72.6%

CI = confidence interval.

Supplementary Figures

Supplementary Fig. 1: Representative chest radiographs and reports of true-positive case.

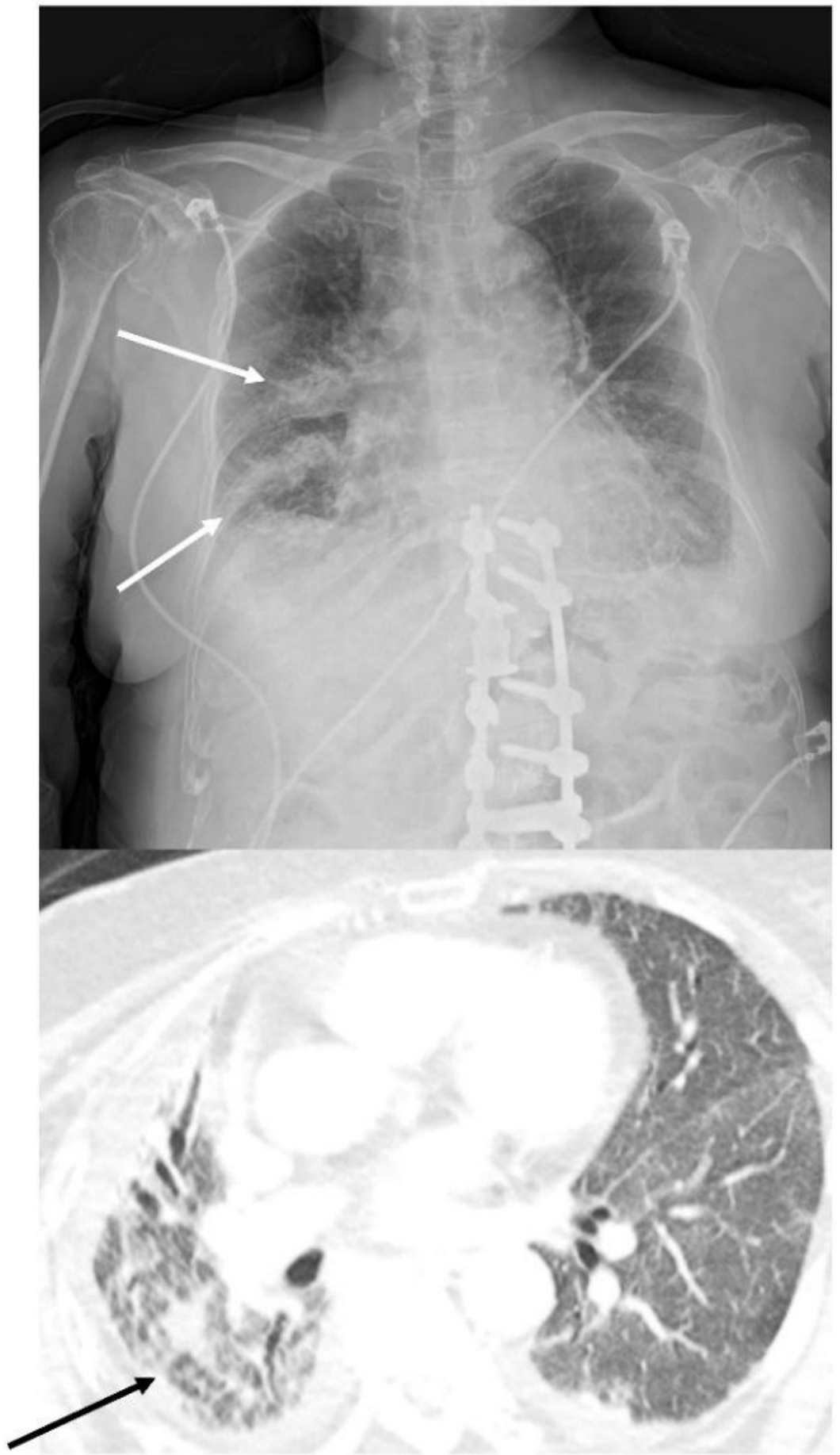

Radiologist:
Diffuse haziness in both lungs.
Consolidation in Rt. lung.

Lingshu:
Findings:
The heart is normal in size. There is no mediastinal widening. Diffuse bilateral airspace opacities are present. No large pleural effusion or pneumothorax.
Impression:
Bilateral airspace opacities may reflect pulmonary edema or infection.

MAIRA-2:
PA AP chest demonstrates cardiomegaly. Bilateral infiltrates are observed right greater than left. There is a right pleural effusion.

AIRead:
Pulmonary edema with both pleural effusion.
DDx. combined pneumonic infiltration.

MedVersa:
Findings:
Single supine AP portable view of the chest was obtained. There are diffuse bilateral pulmonary opacities, right greater than left, with differential diagnosis including pulmonary hemorrhage, pulmonary contusion, infection, or asymmetric pulmonary edema. No large pleural effusion is seen. There is no evidence of pneumothorax. The cardiac silhouette is not enlarged. Mediastinal contours are unremarkable.
Impression:
Diffuse bilateral pulmonary opacities with differential diagnosis including pulmonary hemorrhage, pulmonary contusion, infection, or asymmetric pulmonary edema. No large pleural effusion. No evidence of pneumothorax.

MedGemma:
Okay, here's a description of the chest X-ray based on the image provided:
Overall Impression:
The X-ray shows a significant abnormality in the right lung, with a large opacity/consolidation. There is also evidence of pulmonary edema in the left lung. The heart size appears to be within normal limits.
Detailed Findings:
* **Right Lung:** There is a large area of increased density (opacity) in the right lung, likely representing consolidation, pneumonia, or possibly a mass. The borders of this opacity are not well-defined.
* **Left Lung:** There is evidence of pulmonary edema, which appears as increased interstitial markings and possibly some perihilar haziness.
* **Heart:** The heart size appears to be within normal limits.
* **Mediastinum:** The mediastinum appears unremarkable.
* **Bones:** The visualized bony structures (ribs, clavicles, scapulae) appear intact.
* **Diaphragm:** The diaphragms are seen, but the image quality is not optimal for detailed assessment.
Possible Considerations:
* The large opacity in the right lung needs further evaluation to determine the underlying cause. This could be due to infection (pneumonia), fluid accumulation (pleural effusion or edema), or a mass.
* The pulmonary edema in the left lung could be related to heart failure, kidney disease, or other conditions that cause fluid overload.
Disclaimer:
This is a preliminary interpretation based on a single image. A definitive diagnosis requires a complete clinical history, physical examination, and potentially additional imaging studies (e.g., CT scan).
In summary, the X-ray shows a significant abnormality in the right lung and pulmonary edema in the left lung. Further investigation is needed to determine the underlying cause.

A 74-year-old female with dyspnea. Frontal chest radiograph shows multifocal patchy ground-glass opacities and consolidation, predominantly in the right lung (white arrows), with bilateral costophrenic angle blunting suggestive of bilateral pleural effusion. CT confirmed corresponding abnormalities (black arrow). All report sources appropriately detected lung opacity (with variable terminology); however, bilateral pleural effusion was missed in some reports (radiologist, Lingshu, MedVersa). Green=true positive/true negative; red=false positive/false negative.

Supplementary Fig. 2: Representative chest radiographs and reports of false-positive case.

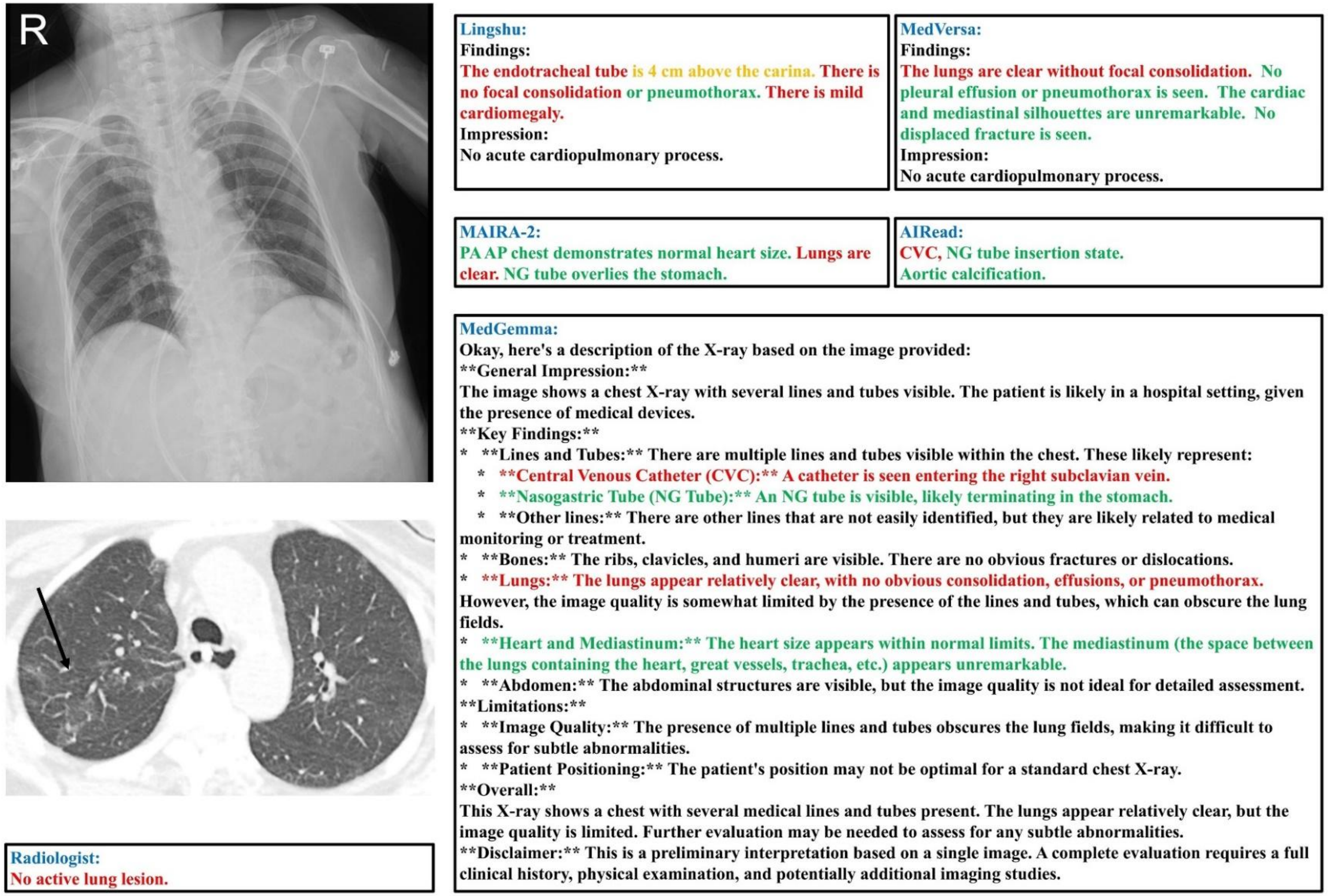

A 66-year-old female with fever. Chest radiograph was interpreted as normal by all readers; CT revealed subtle bilateral ground-glass opacities (black arrow), suggestive of

atypical pneumonia. Lingshu reported endotracheal intubation, which was a false positive. Green=true positive/true negative; red=false positive/false negative; yellow=hallucination.
